# Rethinking the prognostic and biological significance of neuroendocrine marker expression in colorectal carcinoma

**DOI:** 10.3389/fendo.2026.1814001

**Published:** 2026-04-13

**Authors:** Alessandro Mangogna, Giovanna Sabella, Massimo Milione

**Affiliations:** 1 Department of Medicine, Institute of Pathological Anatomy, University of Udine, Udine, Italy; 2 Department of Life Sciences, University of Trieste, Trieste, Italy; 3 1^st^ Pathology Division, Department of Pathology and Laboratory Medicine, Fondazione IRCCS Istituto Nazionale dei Tumori, Milan, Italy

**Keywords:** amphicrine tumours, colorectal cancer, MiNEN (mixed neuroendocrine–non-neuroendocrine neoplasm), neuroendocrine differentiation, synaptophysin

## 1 Introduction

Colorectal carcinoma (CRC) is a biologically heterogeneous disease that has historically been classified according to morphological and immunophenotypic criteria. Neuroendocrine differentiation represents one of the most debated and conceptually unresolved issues in gastrointestinal pathology. The current World Health Organization (WHO) classification defines mixed neuroendocrine–non-neuroendocrine neoplasms (MiNENs) as tumours in which each component constitutes at least 30% of the tumour volume (1, 2). However, this threshold-based definition fails to address a broad diagnostic “grey zone,” encompassing tumours with subthreshold mixed morphology, amphicrine differentiation, and conventional adenocarcinomas exhibiting general neuroendocrine markers (synaptophysin and INSM1) expression in the absence of overt neuroendocrine morphology (3–5).

Recent analyses have identified both conceptual and practical limitations within this framework (3). Although MiNENs and pure neuroendocrine carcinomas (NECs) are consistently associated with aggressive behaviour (6), the biological and prognostic significance of neuroendocrine marker expression in otherwise conventional colorectal adenocarcinomas remains a subject of ongoing debate (4, 6–9).

Building on these observations, neuroendocrine differentiation in CRC should not be regarded as incidental. Instead, accumulating morphological and molecular evidence indicates that this phenomenon may represent biologically significant tumour plasticity with potential prognostic implications.

## 2 Challenges in the interpretation of neuroendocrine differentiations

### 2.1 The diagnostic ambiguity beyond the 30% threshold

The 30% cutoff for MiNEN diagnosis was established to ensure the clinical relevance of both tumour components (1, 2). However, this threshold is arbitrary and lacks robust biological validation. As a result, tumours with biphenotypic morphology that do not meet the 30% criterion are challenging to classify, which may compromise diagnostic consistency and influence patient management (3).

Amphicrine carcinomas, characterized by the coexistence of mucin production and neuroendocrine marker expression within the same tumour cell, further complicate the binary distinction between exocrine and neuroendocrine differentiation (3, 10). The classification of these tumours remains unresolved, and diagnostic reproducibility is hindered by the lack of standardized criteria.

In routine clinical practice, it is necessary to establish clear guidelines to allow the definition of these categories. First and foremost, a careful morphological assessment is required: the presence of even very small and sparsely represented mucinous foci may provide the first indication of an amphicrine neoplasm. Less obvious but equally informative is the evaluation of nuclear features, as amphicrine tumours tend to have vesicular nuclei with prominent nucleoli, more similar to those of adenocarcinomas than to neuroendocrine neoplasms. The most reliable approach, capable of providing a definitive diagnosis on its own, is dual staining with synaptophysin and Alcian-PAS. This technique demonstrates the coexistence of a mucinous or glandular phenotype (stained light blue) and intracellular neuroendocrine components (stained brown or red). Unfortunately, this method is not readily available in all laboratories.

Conventional adenocarcinomas exhibiting diffuse synaptophysin expression in the absence of neuroendocrine morphology also complicate diagnostic interpretation. Some authors have considered this expression incidental and without prognostic significance (4, 7). However, this perspective may underestimate the extent of underlying biological heterogeneity.

Collectively, these challenges demonstrate that rigid categorical systems may obscure an underlying biological continuum rather than accurately representing discrete entities.

### 2.2 Inconsistent evidence regarding prognostic significance

The most reliable prognostic indication has been demonstrated by two manuscripts (11, 12), based on the largest cohort of mixed neoplasms studied by the Italian multicentre, multidisciplinary group. This study relied on a centralised pathological review, which confirmed only 166 cases as true mixed neoplasms (including amphicrine tumours) out of a much larger study population. By comparing mixed neoplasms with pure NECs, the study concluded that in MiNENs the Ki-67 cut-off of 55% is also applicable (for the NEC component only), identifying two distinct populations: those with Ki-67 <55%, who do not respond to platinum-based regimens and have longer survival, and those with Ki-67 ≥55%, who respond to platinum regimens but have very short survival. This work demonstrates that in the <55% category, MiNENs show better survival compared with pure NECs, whereas in the ≥55% category there is no significant difference between MiNENs and NECs (11).

Another seminal work on this topic is by Fassan et al. (13), which demonstrates that the proportion of NEC within the tumour does not significantly influence prognosis. The study shows that even synaptophysin positivity alone is sufficient to significantly shorten survival.

Evidence concerning the prognostic value of general neuroendocrine markers expression in CRC remains inconsistent.

Konukiewitz et al. (4) found that neuroendocrine differentiation in conventional colorectal adenocarcinomas did not significantly influence patient outcomes. Comparable findings have been reported in other studies assessing isolated synaptophysin or chromogranin A expression (9, 14).

In contrast, a recent large cohort study of 663 conventional colorectal carcinomas without neuroendocrine morphology demonstrated that synaptophysin expression in at least 30% of gland-forming tumour cells served as an independent adverse prognostic factor for both overall and disease-free survival (9). This association remained significant in multivariable analyses adjusted for stage and other clinicopathological variables.

Additionally, an amphicrine phenotype was identified in 4.1% of cases, characterized by synaptophysin immunoreactivity and mucin secretion within the same tumour cells (9). The biological identity and clinical significance of these tumours remain undefined.

These discrepancies across studies likely result from variations in cohort size, methodology, positivity thresholds, and inclusion criteria. This variability highlights the urgent need for standardized definitions of clinically meaningful neuroendocrine differentiation.

### 2.3 Molecular evidence and tumour plasticity

Molecular analyses offer critical context for this debate. Colorectal MiNENs and NECs share key driver mutations, including *APC*, *TP53*, and *KRAS*, with conventional adenocarcinomas, supporting a common clonal origin (3, 15). Rather than representing true collision tumours, these neoplasms most likely arise from a single precursor cell capable of divergent differentiation.

This concept is consistent with broader evidence of tumour plasticity and lineage infidelity observed in epithelial cancers (16, 17). Transdifferentiation and dedifferentiation phenomena are documented in colorectal carcinogenesis, particularly under therapeutic pressure (18). Neuroendocrine differentiation may therefore represent a phenotypic shift within the adenocarcinoma lineage rather than a distinct entity.

Consequently, neuroendocrine marker expression may reflect underlying transcriptional reprogramming, which could be associated with aggressive behaviour, stemness characteristics, or treatment resistance.

The central question may not concern whether neuroendocrine differentiation defines a separate tumour category. Rather, it is more informative to consider where a given tumour resides along a biological continuum.

In routine molecular diagnostic practice, large-cell NECs share the same key mutations: *TP53*, *RB1*, and organ-specific mutations such as *APC* in the colon, *KRAS* in the pancreas, and *EGFR* in the lung. We have attempted, through genomic and transcriptomic studies (19–21), to address this question primarily in pulmonary neoplasms, and through a similar genomic and transcriptomic study in gastro-entero-pancreatic (GEP) NECs (22).

The results between lung and GEP NECs are largely overlapping. In particular, in the pulmonary setting, mixed neoplasms composed of adenocarcinoma and large-cell NEC cluster with the corresponding pure neoplasms, whereas mixed adenocarcinoma and small-cell NEC cluster exclusively with small-cell NEC. This finding is consistent with previous literature (23) and supports the hypothesis of a common glandular stem cell from which adenocarcinomas, NECs, and MiNENs may originate. What determines the “choice” of one of these three pathways, however, remains unknown.

### 2.4 Toward an integrated morphological and molecular framework

Current classification systems remain predominantly based on morphological criteria (1, 2). However, emerging molecular insights increasingly challenge the adequacy of purely histology-based stratification.

Future research should aim to achieve the following objectives: a) Develop classification systems that move beyond rigid percentage thresholds; b) Establish reproducible diagnostic criteria for amphicrine carcinomas; c) Define standardized cutoffs for neuroendocrine marker expression that are clinically meaningful; d). Integrate transcriptomic and molecular profiling approaches to identify biologically coherent subgroups.

Large-scale comparative analyses, including RNA sequencing, of conventional adenocarcinomas with neuroendocrine marker expression, amphicrine carcinomas, MiNENs, and NECs may clarify whether these entities constitute distinct molecular subclasses or exist along a continuum within the differentiation spectrum.

Such integration may ultimately refine prognostic stratification and inform therapeutic decision-making.

## 3 Discussion

Neuroendocrine differentiation in CRC challenges the established paradigm in gastrointestinal pathology that separates adenocarcinoma from neuroendocrine carcinoma, as defined by the current WHO classification (1, 2). While this dichotomy has provided practical diagnostic guidance, accumulating morphological, immunophenotypic, and molecular evidence suggests that it does not fully encompass the biological complexity of colorectal tumorigenesis (3).

A central issue in this debate is the conceptual limitation of threshold-based classification. The 30% cutoff for defining MiNENs was introduced to ensure clinical relevance of both components and to avoid overdiagnosis of focal neuroendocrine differentiation (1, 2). However, this threshold lacks robust biological validation. Tumours with clear biphenotypic morphology that do not meet the 30% criterion remain difficult to classify (3), whereas those just above this threshold are labelled as MiNENs despite a lack of evidence for a distinct biological transition. This artificial discontinuity may obscure an underlying biological gradient. Building on this, changes in classification frameworks are further challenged by the emergence of new tumour types.

The growing recognition of amphicrine carcinomas further challenges categorical frameworks. Amphicrine tumours, defined by the coexistence of mucin production and neuroendocrine marker expression within the same tumour cell, defy the traditional separation between exocrine and neuroendocrine lineages (3, 10). Their unresolved taxonomic status underscores the limitations of a strictly morphology-based approach. Rather than being anomalous exceptions, amphicrine carcinomas may exemplify the intrinsic plasticity of colorectal epithelial neoplasia. To deepen this perspective, molecular data offer key supporting evidence.

Molecular data strongly support a continuum-based model. Comprehensive genomic analyses have shown that colorectal MiNENs and NECs share key driver mutations with conventional adenocarcinomas, including alterations in *APC*, *TP53*, and *KRAS* (3, 6). These findings challenge the collision tumour model and instead support a monoclonal origin with subsequent divergent differentiation. In this context, neuroendocrine differentiation is better understood as a phenotypic shift within a single evolving neoplastic clone rather than the coexistence of two independent tumour types. This molecular insight aligns with broader evidence of lineage plasticity in oncologic research.

This interpretation is consistent with broader oncologic evidence that highlights lineage plasticity and transdifferentiation as central mechanisms of tumour evolution and therapeutic resistance (16, 17). Plasticity enables tumour cells to reprogram transcriptional networks in response to intrinsic genomic instability or extrinsic selective pressures, such as systemic therapy. In colorectal cancer, adaptive mutability and phenotypic reprogramming have been implicated in resistance to targeted treatments (18). Thus, neuroendocrine differentiation may represent a manifestation of such plasticity, potentially associated with dedifferentiation, stemness features, or altered proliferative signalling. Shifting focus, the biological significance of marker expression warrants closer consideration.

Within this framework, neuroendocrine marker expression in conventional adenocarcinomas may have biological significance beyond simple immunohistochemical positivity. Although some studies have found no clear prognostic impact of neuroendocrine differentiation in otherwise conventional colorectal carcinomas (4, 7, 8, 14), the present findings indicate that diffuse synaptophysin expression, defined as at least 30% of gland-forming tumour cells, is an independent adverse prognostic factor for both overall and disease-free survival (9). The persistence of this association after multivariable adjustment suggests that neuroendocrine differentiation may reflect biologically aggressive features not fully captured by traditional staging parameters.

Discrepancies among published studies likely result from methodological heterogeneity rather than fundamentally different biological phenomena. Variability in antibody selection, scoring systems, and positivity thresholds can significantly influence reported frequencies and outcomes (4, 7, 8, 14). Furthermore, the lack of standardized criteria for clinically meaningful neuroendocrine differentiation impedes cross-study comparability. Harmonization of immunohistochemical evaluation, potentially through consensus guidelines, is an essential first step toward clarifying the true prognostic impact of these features. Focusing next on amphicrine tumours provides another illustrative example of these complexities.

The amphicrine phenotype requires particular attention. In this cohort, amphicrine carcinomas accounted for approximately 4% of cases (9), yet their biological identity remains poorly defined. If amphicrine tumours demonstrate molecular clustering distinct from both conventional adenocarcinomas and MiNENs, this would support their recognition as a discrete subgroup. Conversely, if transcriptomic profiling places them along a continuum between exocrine and neuroendocrine extremes, this would reinforce the plasticity model. Integrative analyses that combine morphology, immunophenotype, and high-throughput sequencing are therefore essential.

Beyond classification, potential therapeutic implications must be addressed. NECs and MiNENs are managed according to neuroendocrine carcinoma protocols (1, 2). However, no established management strategy exists for conventional adenocarcinomas with diffuse neuroendocrine marker expression or amphicrine features. If future studies reveal shared transcriptional programs or therapeutic vulnerabilities between these tumours and NECs, treatment paradigms may require revision. Alternatively, if these tumours cluster molecularly with conventional adenocarcinomas, their prognostic impact may reflect intrinsic biological aggressiveness rather than distinct therapeutic sensitivity. Against this background, intratumoral heterogeneity emerges as an additional area for exploration.

Intratumoral heterogeneity introduces additional complexity. Neuroendocrine differentiation may be spatially heterogeneous, raising concerns about sampling bias in limited biopsy specimens. Subclonal neuroendocrine populations may also expand under therapeutic pressure, similar to plasticity phenomena observed in other tumour types (16, 17). Longitudinal studies that include matched primary and metastatic samples, as well as pre- and post-treatment comparisons, are necessary to elucidate the temporal dynamics of neuroendocrine differentiation in colorectal cancer. These classification and conceptual questions lead to the field’s central challenge.

Ultimately, the challenge is both classificatory and conceptual. The question arises whether tumour taxonomy should impose rigid categorical distinctions or evolve toward models that reflect biological continua. The progression of colorectal cancer classification from purely morphological systems to the integration of molecular subtypes demonstrates that taxonomy adapts to emerging biological knowledge. Accumulating evidence of shared clonal origins (3, 6, 15), plasticity mechanisms (16–18), and potential prognostic impact (9) suggests that neuroendocrine differentiation represents a spectrum within colorectal tumour evolution rather than a strictly separate entity.

**Figure 1:**
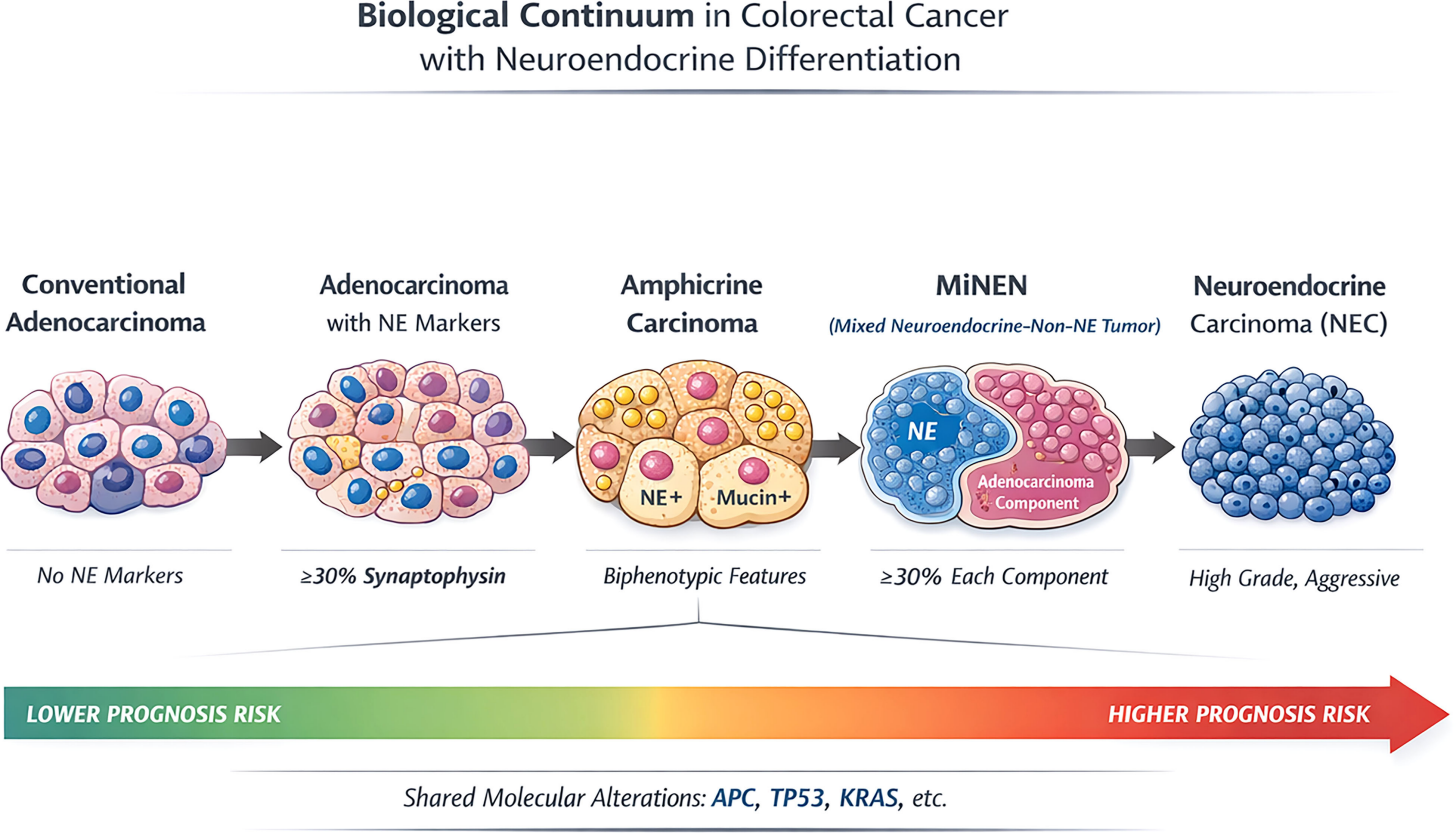
Conceptual model illustrating the continuum of neuroendocrine differentiation in colorectal carcinoma. The proposed biological continuum of neuroendocrine differentiation in colorectal cancer demonstrates that conventional colorectal adenocarcinoma may progressively acquire neuroendocrine characteristics. This progression includes focal or diffuse neuroendocrine marker expression without morphological changes, amphicrine differentiation characterized byco-expression of mucin and neuroendocrine markers within individual tumor cells, mixed neuroendocrine–non-neuroendocrine neoplasms (MiNENs), and ultimately pure high-grade neuroendocrine carcinomas (NECs). These categories do not represent strictly distinct entitiesdefined by rigid percentage thresholds; instead, they reflect progressive phenotypic plasticity along a shared clonal trajectory. The continuum further correlates with increasing biological aggressiveness and dedifferentiation. This continuum should be envisioned as a series of potentially concurrent events acting on a glandular stem cell, leading – through different timingand mechanisms – to the development of distinct neoplastic categories, each differing in morphology and prognosis.

As illustrated in **Figure 1**, a continuum-based model is proposed in which conventional adenocarcinoma, adenocarcinoma with diffuse neuroendocrine marker expression, amphicrine carcinoma, MiNEN, and NEC represent progressive differentiation states along a shared biological axis. Recognizing this continuum does not diminish the practical utility of current classifications but encourages refinement through the integration of molecular and clinical data.

In conclusion, neuroendocrine differentiation in colorectal cancer should neither be dismissed as incidental nor prematurely classified as a distinct tumour type in all contexts. Instead, it likely represents a dynamic expression of tumour plasticity with potential prognostic and therapeutic relevance. Constructive interdisciplinary collaboration and harmonized methodological approaches are essential to resolve remaining ambiguities and to translate biological insights into clinically meaningful stratification.

The proposed biological continuum of neuroendocrine differentiation in colorectal cancer demonstrates that conventional colorectal adenocarcinoma may progressively acquire neuroendocrine characteristics. This progression includes focal or diffuse neuroendocrine marker expression without morphological changes, amphicrine differentiation characterized by co-expression of mucin and neuroendocrine markers within individual tumour cells, mixed neuroendocrine–non-neuroendocrine neoplasms (MiNENs), and ultimately pure high-grade neuroendocrine carcinomas (NECs). These categories do not represent strictly distinct entities defined by rigid percentage thresholds; instead, they reflect progressive phenotypic plasticity along a shared clonal trajectory. The continuum further correlates with increasing biological aggressiveness and dedifferentiation. This continuum should be envisioned as a series of potentially concurrent events acting on a glandular stem cell, leading – through different timing and mechanisms – to the development of distinct neoplastic categories, each differing in morphology and prognosis.
